# Two Acute Cases

**Published:** 1895-02

**Authors:** C. L. Olds

**Affiliations:** Philadelphia


### TWO ACUTE CASES.

C. L. OLDS, M. D., H. M., PHILADELPHIA.

July 10th, 1893.—Miss Marie L——, age twenty. Colic that makes her double up and scream. Paroxysms about every five minutes; most agonizing pain.

She had been eating a large amount of ice-cream the day previous. A physician had given her Ars.$^{30}$ in water, but it did no good.

Extremely cranky; cannot give a civil answer to my questions. The "bite-your-head-off" disposition.

Chilly; has many covers, although it is quite warm.

Diarrhœa; stool in small quantities and frequent; yellowish, thin, slimy. *Nux-v.*$^{cm}$ (F. C.), one dose.

The next paroxysm of colic was much lighter and the last that she had.

The diarrhœa soon came on more profusely, but lasted only one day longer.

July 13th, 1894.—Mrs. C——, age forty. Was called in haste to see her. Husband said that she was having terrible pains in the abdomen.

She had had a bad diarrhœa that day and was advised to take "blackberry brandy." She had taken about four ounces of the brandy at one dose, which stopped the diarrhœa.

Lying on bed with flushed face and dilated pupils.

Colicky pains in abdomen, causing her to bend double and scream at the top of her voice and thrash about the bed. Paroxysms every two minutes. She seemed in awful agony.

Amelioration from hard pressure on abdomen. *Coloc.*$^{cm}$ (F. C.), one dose and S. L.

In fifteen minutes she was quiet and sleeping.

These cases are reported, not because they are unusual, but because they are plain, ordinary cases, such as are encountered almost every day. It is in these violent, acute attacks that the mongrel, who is always more or less of a routinist, uses his hypodermic upon the patient who does not at once respond to his repeated doses of the lowest dilutions or triturations. Here it is, in these common, acute affections, that patients expect Homœopathy to relieve them. If a few sugar pellets on the tongue will stop that awful pain, they will believe in Homœopathy and in their doctor, but if they see their physician using a hypodermic, what reason have they for thinking that his system is above that of the allopath? It is true that a dose of Morphine will suppress the pains of the sufferer, will put him into a quiet sleep, and, moreover, will hush the friends of the patient, making them believe that something is being done. Too many doctors make their prescriptions to hush the cry and clamor of the attendants, rather than for the ultimate good of the patient. These doctors are afraid of the attendants, and are not masters of the situation.

Besides, who dare assert that Morphine will quiet the patient and stop those awful pains more quickly than the similar remedy in potentized form? Who, that has faithfully followed the precepts laid down in *The Organon*, can doubt that the similar remedy is more powerful to assuage pain than a hypnotic? And which patient will be in the better condition the next day, the one that had the hypodermic or the one that had the similar remedy?

